# Embryology of *Anoectochilus roxburghii*: seed and embryo development

**DOI:** 10.1186/s40529-019-0254-1

**Published:** 2019-04-22

**Authors:** Yuan-Yuan Li, Zhi-Xia Meng, Ying Zhang, Shun-Xing Guo, Yung-I Lee

**Affiliations:** 1 Institute of Medicinal Plant Development, Chinese Academy of Medical Sciences & Peking Union Medical College, Beijing, 100193 People’s Republic of China; 2 Biology Department, National Museum of Natural Science, No 1, Kuan-Chien Rd, Taichung, 40453 Taiwan; 3 Department of Life Sciences, National Chung Hsing University, Taichung, 40227 Taiwan

**Keywords:** Embryogenesis, Jewel orchid, Seed coat, Suspensor

## Abstract

**Background:**

*Anoectochilus roxburghii* is known for its medicinal properties, culinary interests, and ornamental applications in Asian countries. Recent studies focus mainly on its phytochemical properties and little is known about its reproductive biology, especially seed and embryo development. This study documents the major developmental events in seed and embryo development of *A. roxburghii* upon pollination.

**Results:**

Morphological and histological studies revealed that upon pollination embryo and seed development is completed in 40 days. Ovular primordia are at the megaspore mother cell stage at the time of anthesis. Embryo development proceeds after a successful fertilization. *A. roxburghii* has a single cell suspensor. It elongates but not extended beyond the seed coat. A distinct cell gradient is present within the embryo proper with smaller cells located towards the chalazal end of the seed. Proteins and lipids are the major storage products within the embryo proper cells. At the stage of early globular embryo, the inner seed coat has degenerated and thus a carapace is absent at maturity. A limited deposition of lignin is detected in the mature seed coat.

**Conclusions:**

The seed of *A. roxburghii* matures rapidly. At maturity, the embryo proper has a well-differentiated apical zone with little constraints impose by the seed coat. These characters indicate adaptations to fast germination that may ensure a successful colonization in the shaded forest understory.

**Electronic supplementary material:**

The online version of this article (10.1186/s40529-019-0254-1) contains supplementary material, which is available to authorized users.

## Background

The genus *Anoectochilus*, commonly known as marbled jewel orchids, is a small terrestrial orchid in subtropical and tropical regions. It comprises about 60 species native to habitats ranging from the Himalayas to south China and southeast Asia, Australia, New Guinea and Melanesia (Pridgeon et al. 2003; Govaerts 2018). *Anoectochilus roxburghii* has medicinal, culinary, and ornamental applications in several Asian countries (Chen et al. 2009). In traditional medicine, the whole plant can be used for heat dissipation, elimination of dampness, detoxification, and immunity enhancement (Ye et al. 2017). *A. roxburghii* likes to grow in the humus-rich soil under broad leaf and evergreen primary forests at elevations of 300–800 m above sea level. During October to December, the flower spikes are up to 25 cm tall and each spike produces 2–6 flowers near 1 cm in diameter (Chen et al. 1999). Nowadays, because of huge market demands, wild populations of *A. roxburghii* have decreased sharply due to over-collection.

Similar to other orchid species, the tiny *A. roxburghii* seed has a rudimentary embryo and lacks endosperm (Arditti and Ghani 2000; Yam et al. 2002). Seed germination requires mycorrhizal association, which supplies nutrients for the germinating seed until the seedling develops green leaves and becomes autotrophic (Rasmussen 1995). Until now information of reproductive development in orchid species in the subtribe Goodyerinae is limited. The objectives of this study were to document key developmental and anatomical events in the embryogenesis of *A. roxburghii*. Besides morphological characterization, we used the Historesin embedding method to provide high quality serial sections to examine developmental events during the course of seed development. The observation provides essential knowledge for future investigations into the reproductive biology of *A. roxburghii*.

## Methods

### Plant materials

**Fig. 1:**
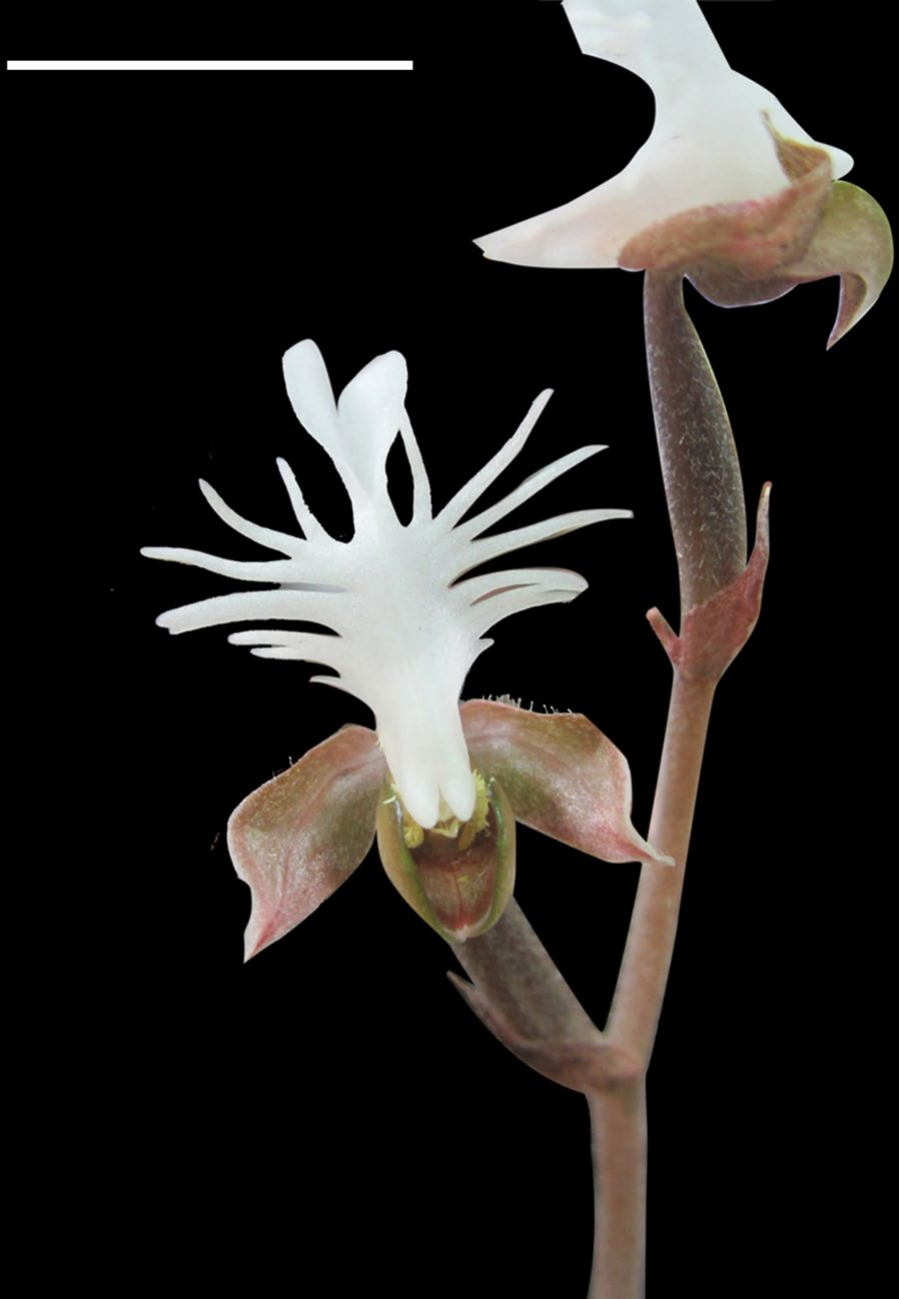
The flower of *A. roxburghii*. Scale bar = 1 cm

Plants of *A. roxburghii* were grown in the greenhouse at the Institute of Medicinal Plant Development, Chinese Academy of Medical Sciences & Peking Union Medical College, Beijing, China. Blooming of *A. roxburghii* usually occurred from October to December. To guarantee a good capsule set and seed quantity, flowers were hand-pollinated at the time of anthesis (Fig. 1). Developing capsules were harvested at regular intervals after pollination. Around 90 developing capsules were harvested for this study.

### Light microscopy and histochemical observations

Development capsules were sliced and fixed immediately with 2.5% glutaraldehyde in 0.1 M phosphate buffer, pH 6.8 at room temperature for 6 h. After fixation, the samples were dehydrated using an ethanol series, and embedded in Technovit 7100 (Kulzer & Co., Germany) as described by Yeung and Chan (2015). Serial, 3 µm-thick sections were cut using glass knives by a Reichert-Jung 2040 Autocut rotary microtome. These sections were collected on slides and stained with Periodic acid–Schiff’s reaction for total insoluble carbohydrates, and counterstained with either 0.05% (w/v) toluidine blue O (TBO) in the benzoate buffer for general histology or 1% (w/v) amido black 10B in the 7% acetic acid solution for protein staining (Yeung 1984). The presence of cuticular material was stained using Nile red as described by Lee et al. (2006). The sections were stained with 1 μg ml^−1^ of Nile red (Sigma Chemical Co., St. Louis, Mo.) for 3 min, briefly washed in distilled water for 1 min, and mounted in the Vectashield^®^ anti-fading medium (Vector Laboratories, Inc., Burlingame, CA). The fluorescence signal was examined using an epifluorescence microscope (Axioskop 2, Carl Zeiss AG) equipped with the Zeiss filter set. The images were captured digitally using a CCD camera attached to the microscope.

### In vitro seed germination

The mature seeds were collected from capsules and surface sterilized with a 0.5% sodium hypochlorite solution with 0.1% wetting agent (Tween 20) for 15 min. After sterilization, the seeds were rinsed three times in sterile distilled water. The seeds were inoculated onto the 1/4 Murashige and Skoog (MS) medium (Murashige and Skoog 1962), supplemented with 2 mg l^−1^ glycine, 0.5 mg l^−1^ niacin, 0.5 mg l^−1^ pyridoxine HCl, 0.1 mg l^−1^ thiamine, 1 g l^−1^ tryptone, 20 g l^−1^ sucrose, 100 ml l^−1^ coconut water and solidified with 7 g l^−1^ agar (Sigma-Aldrich Co.). The pH of the medium was adjusted to 5.6 before autoclaving at 121 °C for 15 min. After sowing, the cultures were incubated in the dark at 25 ± 1 °C. Each culture tube was examined at 15-day intervals for 60 days in culture under a stereomicroscope. Germination was considered as emergence of the embryo from the testa.

## Results

**Fig. 2:**
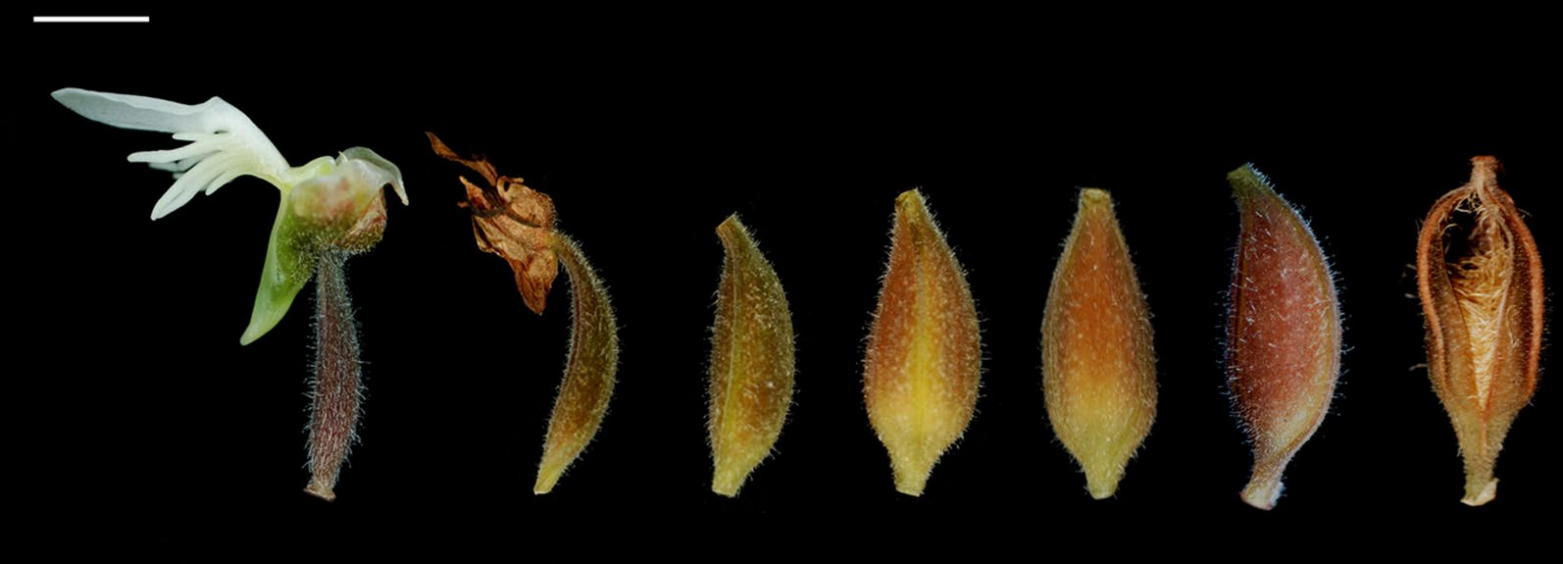
Light micrographs of developing capsules of *A. roxburghii*. From left to right, 0, 5, 10, 15, 20, 30 and 40 DAP. Scale bar = 5 mm

Table 1 describes major developmental events and changes in structure, size and color of capsules and seeds of *A. roxburghii* from pollination to seed maturity. The un-pollinated ovary was dark reddish-green in color covered with epidermal hairs. Upon a successful pollination, flowers senesced quickly and ovaries began to enlarge and turned into capsules (Fig. 2). As the capsules enlarged, the color turned into light reddish-green to light reddish-brown from 5 to 20 days of pollination (DAP). As the capsules approached maturity, the color became reddish brown at 30 DAP, and the capsules split to release seeds at 40 DAP.

**Table 1:** Major developmental events occurring in developing capsules of *A. roxburghii* after fertilization

DAP	Developmental stage	Capsule size (mm)		Capsule color	Seed size (µm)		Seed color
		X-axis	Y-axis		X-axis	Y-axis	
0	Megaspore mother cell	2.36 ± 0.02	12.71 ± 0.03	Dark reddish green	59.4 ± 7.4	163.9 ± 10.2	White
5	Mature embryo sac	2.91 ± 0.05	13.54 ± 0.02	Light reddish green	62.6 ± 6.9	268.6 ± 12.1	White
10	Fertilization and zygote	3.69 ± 0.06	13.43 ± 0.08	Light reddish green	74.2 ± 10.7	583.3 ± 9.4	White
15	Proembryo	3.81 ± 0.08	13.62 ± 0.09	Light reddish brown	85.3 ± 7.4	1085.7 ± 16.5	White
20	Early globular to globular embryo	3.94 ± 0.11	13.81 ± 0.07	Light reddish brown	89.5 ± 14.1	1732.2 ± 22.9	Yellowish white
30	Late globular embryo, and the suspensor starts to degenerate	4.12 ± 0.12	13.75 ± 0.11	Reddish brown	89.9 ± 12.5	2015.1 ± 29.2	A mixture of yellowish white and light brown seeds
40	Mature seed	3.92 ± 0.06	13.48 ± 0.08	Reddish brown	87.1 ± 14.1	2064.5 ± 35.2	Light brown

DAP, days after pollination

**Fig. 3:**
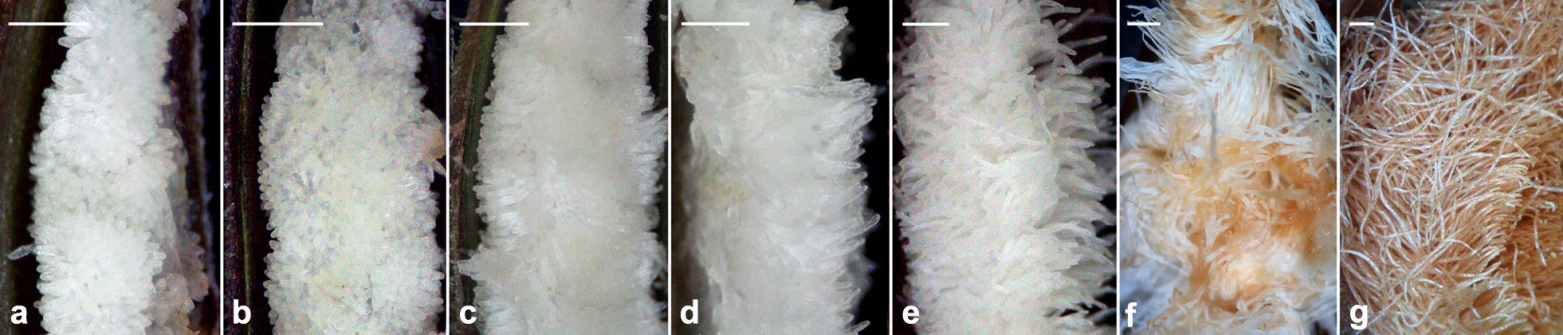
Light micrographs of developing ovules and seeds of *A. roxburghii*. **a** 0 DAP, **b** 5 DAP, **c** 10 DAP, **d** 15 DAP, **e** 20 DAP, **f** 30 DAP and **g** at 40 DAP. Scale bar = 0.5 mm

At the time of anthesis, ovular primordia were well developed within ovaries (Fig. 3a). Upon pollination, the primordia elongated rapidly (Fig. 3b–e). After fertilization, the seed coat continued to elongate and the color of seeds turned from white to yellow (Fig. 3e, f). As the seeds approached maturity at 40 DAP, the hair-like seeds became light-brown and desiccated (Fig. 3g). Seed maturation took 40 days from the time of pollination (Table 1).

**Fig. 4:**
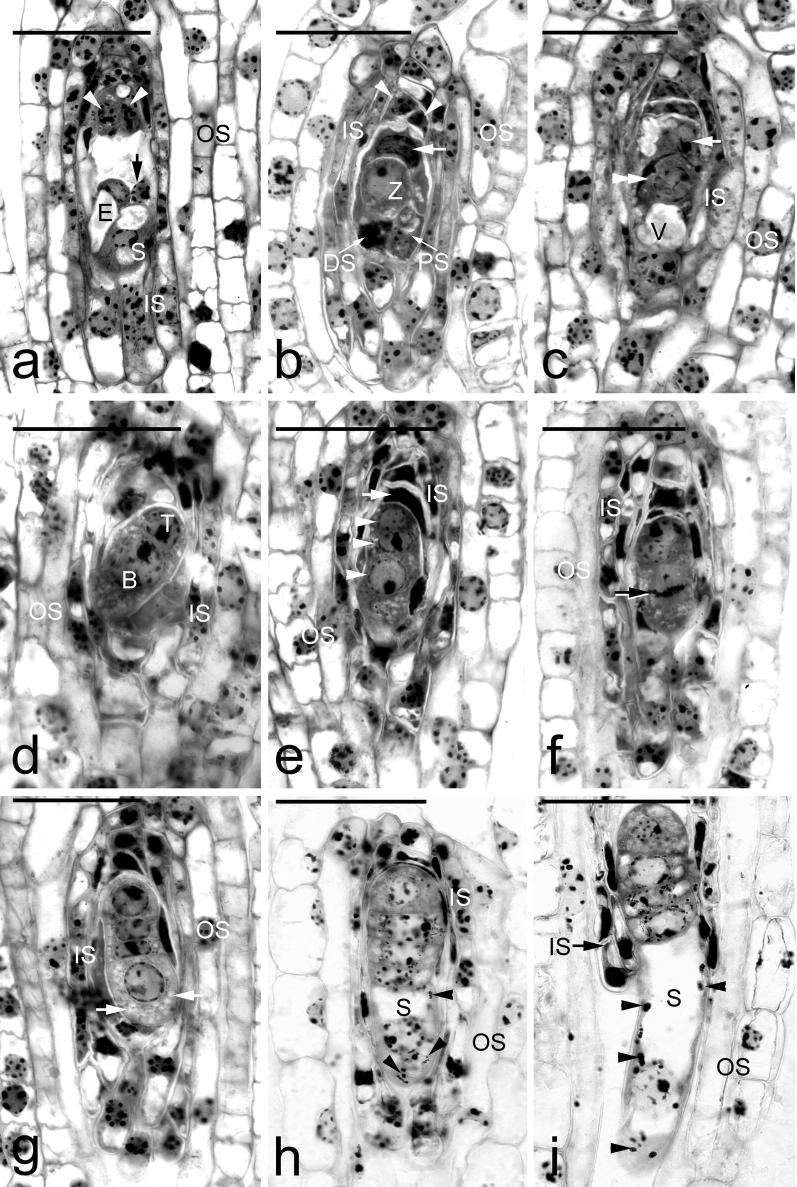
Light micrographs of embryo development of *A. roxburghii* at the early stages. **a** Light micrograph of a mature embryo sac before fertilization at 5 DAP. The egg cell (E) has an elongated shape with a prominent vacuole toward the micropylar end. Antipodal cells (arrowheads), inner seed coat (IS), outer seed coat (OS), polar nucleus (arrow), synergid (S). Scale bar = 30 µm. **b** Light micrograph of the zygote (Z) after fertilization at 10 DAP. At the micropylar end, one synergid has degenerated (DS), and the other synergid is still persistent (PS). The primary endosperm nucleus (arrow) could be observed within the endosperm cavity, but the endosperm eventually fails to develop. Antipodal cells (arrowheads), inner seed coat (IS), outer seed coat (OS). Scale bar = 30 µm. **c** An elongated zygote (double arrowhead) with a prominent vacuole (V) toward the micropylar end. No additional division of the endosperm nucleus (arrow) is observed. Inner seed coat (IS), outer seed coat (OS). Scale bar = 30 µm. **d** The first cell division of the zygote produces a smaller terminal cell (T) and a larger basal cell (B). Inner seed coat (IS), outer seed coat (OS). Scale bar = 30 µm. **e** Light micrograph showing a three-celled embryo (arrowheads), and the endosperm nucleus (arrows) has become condensed. Inner seed coat (IS), outer seed coat (OS). Scale bar = 30 µm. **f** The alignment of metaphase chromosomes (arrow) indicates a transverse division occurring in the basal cell of a three-celled embryo. Inner seed coat (IS), outer seed coat (OS). Scale bar = 30 µm. **g** A four-celled embryo resulting from the transverse division of the basal cell. The cell towards the micropylar end has become larger with a prominent nucleus and numerous small vacuoles (arrows). The cell accumulates numerous small vacuoles in preparation for the further cell elongation. Inner seed coat (IS), outer seed coat (OS). Scale bar = 30 µm. **h** Light micrograph showing a filamentous-shaped embryo. The suspensor cell (S) with a prominent vacuole has enlarged and elongated toward the micropylar end. A few tiny starch granules (arrowheads) are present in the suspensor cell. Inner seed coat (IS), outer seed coat (OS). Scale bar = 30 µm. **i** The suspensor cell (S) elongates rapidly with numerous starch granules (arrowheads) and has protruded into the cavity enclosed by the outer seed coat (OS). At this stage, the inner seed coat (IS) is degenerating (arrow). Scale bar = 30 µm

Ovule development had begun during flower development. At the time of anthesis, the ovular primordia were at the megaspore mother cell stage. It took only 40 days for an ovule to develop into a mature seed (Fig. 4). At 5 DAP, the megaspore mother cell enlarged and differentiated into an embryo sac (Fig. 4a). Fertilization occurred approximately 10 DAP, and embryo development soon commenced. The zygote initially had an ovoid shape (Fig. 4b), then it elongated and became highly polarized (Fig. 4c). The first cell division of the zygote was unequal, producing a smaller terminal cell and a larger basal cell (Fig. 4d). The terminal cell formed the embryo proper while the basal cell gave rise to the suspensor. Moreover, derivatives from the basal cell also contributed cells to the embryo proper. The endosperm failed to develop in this species. After fertilization, the nuclei within the primary endosperm cell did not undergo further division (Fig. 4b). The content of the cell was eventually absorbed by the expanding embryo.

### Suspensor development

**Fig. 5:**
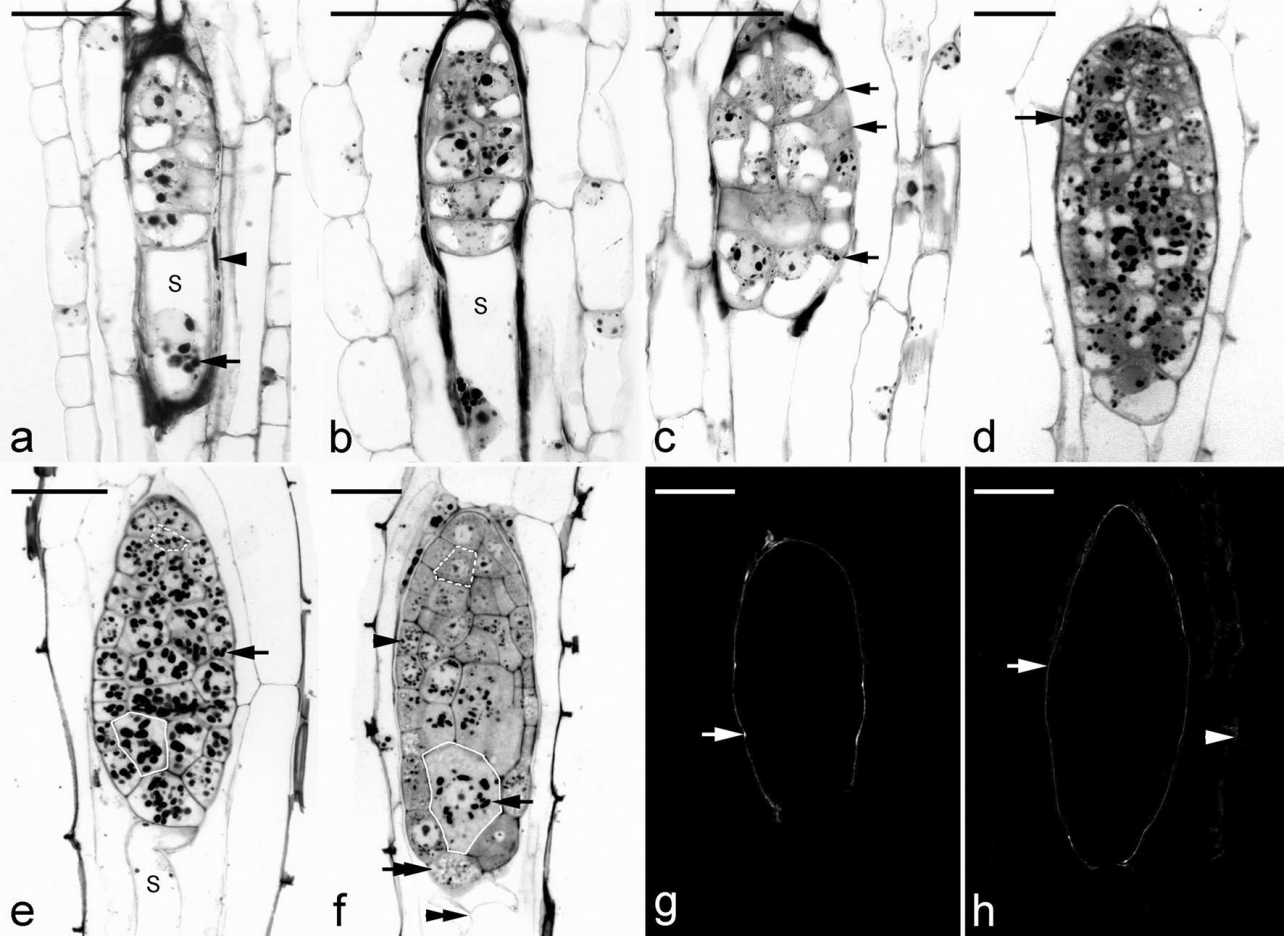
Light micrographs of embryo development of *A. roxburghii* at the late stages. **a** The cell at the terminus divides vertically and increases in size in preparation for further divisions. The suspensor (S) continues to elongate and a few starch grains (arrow) accumulate around the nucleus. At this stage, the inner seed coat has degenerated (arrowhead) and has been resorbed by the developing embryo. Scale bar = 30 µm. **b** The suspensor has elongated further, but it never extends beyond the lumen enclosed by the outer seed coat. Scale bar = 30 µm. **c** Additional periclinal and anticlinal divisions within the embryo proper that result in the formation of the inner tier of cells and the protoderm (arrows). Scale bar = 30 µm. **d** Light micrograph showing an early globular embryo with a discernible protoderm. Numerous starch grains (arrow) accumulate within the cells of embryo proper. Scale bar = 30 µm. **e** As the globular embryo approaches maturity, more starch grains (arrow) accumulate within the embryo proper and the suspensor (S) is going to degenerate. Within the embryo proper, the smaller cell toward the chalazal end is marked by the dashed line and the larger cell toward the micropylar end is marked by the solid line. Scale bar = 30 µm. **f** A longitudinal section through a mature seed. Within the embryo proper, the smaller cell toward the chalazal end is marked by the dashed line and the larger cell toward the micropylar end is marked by the solid line. The suspensor cell (double arrowhead) became dehydrated and finally collapsed. At this stage, the starch grains (arrow) have most disappeared, and numerous small protein bodies (arrowhead) can be seen within the embryo proper. Although the lipid cannot be preserved in this historesin, the spaces between the protein bodies could be the storage lipid bodies (double arrow). Scale bar = 30 µm. **g** Nile red staining fluorescence micrograph of an early globular embryo at the same stage as that seen in **c**. The surface wall (arrow) of the embryo proper reacts positively to the stain, and the fluorescence is absent in the suspensor wall. Scale bar = 30 µm. **h** Nile red staining fluorescence micrograph of a globular embryo at the same stage as that seen in **e**. The surface wall (arrow) of the embryo proper reacts positively to the stain, and seed coat (arrowhead) only reacts weakly. Scale bar = 30 µm

*Anoectochilus roxburghii* has only a single suspensor cell and it is elongated as it matured. The cell lineage resulting in suspensor formation is described as follows. An additional transverse cell division occurred in the two-celled embryo resulting in the formation of a three-celled embryo (Fig. 4e). This was soon followed by a transverse division occurring in the larger basal cell (Fig. 4f), giving rise to a four-celled embryo (Fig. 4g). The micropylar basal cell enlarged in size and destined to become the suspensor (Fig. 4g). When compared to the other three cells toward the chalazal end, the micropylar basal cell was more cytoplasmic with many small vacuoles present. This micropylar basal cell elongated rapidly by the process of vacuolation (Fig. 4h, i). It is notable that starch granules were present in the suspensor cell but less abundant in the embryo proper at this time (Fig. 4h, i). The suspensor cell continued to elongate and finally extended beyond the micropylar opening of the inner seed coat and grew into the lumen enclosed by the outer seed coat (Fig. 4i). However, the suspensor cell never extended beyond the outer seed coat. As the embryo matured, the suspensor cell became dehydrated and finally collapsed (Fig. 5f). The pattern of Nile red staining indicated that a cuticular substance was absent over the walls of the suspensor cell through its development and maturation (Fig. 5g, h).

#### Embryo proper development

In the four-celled embryo, the three cells towards the chalazal end were responsible for the formation of the embryo proper (Fig. 4g). The cells at the terminus of the filamentous embryo began to divide vertically and these newly formed cells enlarged in preparation for further divisions (Fig. 5a). Additional periclinal and anticlinal divisions occurred within the embryo proper, resulting in the formation of an inner tier of cells and the protoderm (Fig. 5c). A distinct protoderm layer was found at approximately 20 DAP, and a number of starch grains had accumulated within the cells of embryo proper (Fig. 5d). As the seed approached maturity (30 DAP), starch grains became more abundant (Fig. 5e).

At maturity (40 DAP), the ellipsoidal embryo was only seven to eight cells along its long axis and four cells across. The cells were of different sizes. The cells toward the chalazal end were smaller than those toward the micropylar end (Fig. 5f). Within the cells of a mature embryo proper, only a few starch grains could be observed; protein and lipid bodies became the major storage products. Nile red staining indicated the presence of cuticular substance in the wall appeared at the early globular stage of embryo development (Fig. 5g). The same staining pattern persisted through embryo maturation, and the staining intensity did not increase as the embryo matured (Fig. 5h).

#### Seed coat development

After fertilization, the inner and outer integuments became the seed coat enclosing the developing embryo (Fig. 4b–g). During the early stages of embryo proper formation, the cells of the inner seed coat progressively degenerated (Fig. 4h–i), and their cell content was apparently absorbed by the developing embryo (Fig. 5a). At the early globular stage, the outer seed coat was two cells thick, and the cell walls of the outermost layer of the seed coat stained purple with the TBO stain, indicating the absence of phenolic compounds in the wall (Fig. 5c). In addition, Nile red staining gave no fluorescence signal from the walls of the outer seed coat (Fig. 5g). As the seed approached maturity, the cells of the seed coat became dehydrated and compressed into a thin layer (Fig. 5e–f). The radial walls of the outermost layer of the seed coat gave a greenish blue color when stained with TBO, indicating the presence of phenolic compounds in the wall (the lignification of cell wall). In addition, the secondary walls reacted weakly to Nile red stain (Fig. 5h).

#### In vitro seed germination

At 15 days after inoculation, the embryo started to enlarge and protrude from the seed coat on 1/4 MS medium. Seed germination reached 72.5% after 60 days of inoculation (Additional file 1: Figure S1).

## Discussion

As shown in Table 1, embryo development and seed maturation in *A. roxburghii* is rapid compared to many orchid species. One of the main reasons is that ovule development commences prior to pollination. In most orchids, ovule development is delayed and triggered by pollination (Yeung and Law 1997). The orchids with a relative rapid ovule development, e.g. *Epipogium aphyllum* (Afzelius 1954), *Epipactis papillosa* (Sato 1974) and *Gastrodia elata* (Kusano 1915) usually have megaspore mother cells and/or embryo sacs present within the ovary at the time of anthesis. In *A. roxburghii*, megaspore mother cells could be observed within the ovary at the time of anthesis, and mature embryo sacs are present at 5 DAP (Additional file 2: Figure S2). It is noteworthy that many orchids with a rapid process of embryo development are terrestrial species occurring in the shaded forest understorey. A rapid seed maturation may ensure a rapid seed dispersal, and thus a successful colonization strategy when growing in the shaded forest understorey.

Most orchids have rudimentary embryos and without a defined tissue pattern. Generally, only a protoderm has differentiated, and depending on the species, a gradient of cell sizes within the embryo proper can be seen with smaller cells located at the chalazal end, denoting the future shoot pole (Andronova 2006). The presence of a well-differentiated apical zone could provide not only an indication of structural differentiation but also an indirect indication on the difficulty in seed germination. In the easy-to-germinate species, such as *Epidendrum* (Yeung 2017), *Phalaenopsis* (Lee et al. 2008) and *Anoectochilus* in this study, a marked gradient of cell size exits in the embryo proper of their mature seeds. While in the difficult-to-germinate species such as *Calanthe* (Lee et al. 2007), *Calypso* (Yeung and Law 1992) and *Cypripedium* (Lee et al. 2005), their embryo proper has cells of similar sizes. The gradient of cell sizes reveals the existence of physiological differences along the embryo in an apical-basal manner. The well-differentiated apical zone within the embryo proper may accelerate the differentiation in shoot apical meristem within a protocorm upon seed germination in asymbiotic cultures (Lee et al. 2013; Yeung 2017). It is also interesting to note the formation of large embryo cells near the micropylar end (Fig. 5e, f). This portion of embryo proper are programmed to house the symbiont in germination. During embryo development, the suspensor cell wall and the cell wall of the large cell adjacent to the suspensor cell are free of cuticle coverage. As the suspensor degenerated at seed maturity, the large cell at the basal end provides a ready entry point for the penetration of mycorrhizal fungi (Jiang et al. 2015). In addition, the persistence of starch grains in the larger cells (Fig. 5f) may serve as an enticement for fungal hyphae.

In developing seeds of orchids, the accumulation of lignin and/or cuticular materials in different layers of the seed coat has been reported in a number of orchids, such as *Apostasia* (Nishimura and Tamura 1993), *Calanthe* (Lee et al. 2007), *Cephalanthera* (Yamazaki and Myoshi 2006), *Cymbidium* (Yeung et al. 1996), *Cypripedium* (Lee et al. 2005), *Cyrtosia* (Yang and Lee 2014), *Paphiopedilum* (Lee et al. 2006) and *Vanilla* (Nishimura and Yukawa 2010). The differences in accumulation of lignin and cuticular materials may affect the seed germination in vitro (Yeung et al. 2018). In those difficult-to-germinate species, the inner integument usually forms a thin layer of inner seed coat, termed ‘carapace’ tightly enclosing the embryo, such as *Cephalanthera* (Yamazaki and Myoshi 2006), *Cypripedium* (Lee et al. 2005) and *Dactylorhiza* (Rasmussen 1995). The thickness of the carapace and the accumulation of cell wall materials, e.g. lignin and/or cuticular materials can be diverse among orchid species. Lignification and cutinization could strengthen the cell walls of seed coat and thus protect the minute embryo at the time of seed dispersal. But the tightly fitted coating forms a physical barrier restricting embryo growth (Miyoshi and Sato 1997). On the other hand, for the easy-to-germinate species (especially the epiphytic orchids), such as *Phalaenopsis* (Lee et al. 2008), the inner seed coat degenerates soon after fertilization, and the lignification only occurs at the radial wall of seed coat forming a discontinuous layer covering the embryo. Seed germination of *Anoectochilus* species is not recalcitrant (Additional file 1: Figure S1, Chou and Chang 2004) as compared to a majority of temperate terrestrial orchids (Lee et al. 2005; Yamazaki and Myoshi 2006). This is likely due to the absence of a distinct carapace. Histochemical staining results indicated an absence of cuticular material in the seed coat and a limited deposition of lignin. These characters may enable the embryo of *A. roxburghii* to access water and nutrients from the environment. Furthermore, there is a less physical restriction to subsequent seed enlargement and germination.

## Conclusions

The developmental timetable summarizes major developmental events in seed and embryo development of *A. roxburghii* upon pollination. At maturity, the marked gradient of cell size in the embryo proper may accelerate the differentiation and formation of a shoot apical meristem within a protocorm upon germination. Together with little constraints impose by the seed coat, *A. roxburghi* seeds germinate readily. The information provided in this study serve as a quick handy reference for future in vivo and in vitro studies of embryo development and seed germination.

## Additional files

**Additional file 1: Figure S1.** In vitro seed germination rate of *A. roxburghii* on 1/4 MS medium. Error bars represent SE (n = 3).

**Additional file 2: Figure S2.** In *A. roxburghii*, the megaspore mother cell (M) could be observed within the ovary at the time of anthesis (A), and the mature embryo sac is present at 5 DAP (B). Egg (E), polar nucleus (P), synergids (S). Scale bar = 50 µm.

## References

[CR1] Afzelius K (1954). Embryo-sac development in *Epipogium aphyllum*. Svensk Botany Tidskr.

[CR2] Andronova EV, Batygina TB (2006). Embryogenesis in Orchidaceae. Embryology of flowering plants.

[CR3] Arditti J, Ghani AKA (2000). Numerical and physical properties of orchid seeds and their biological implications. New Phytol.

[CR4] Chen SC, Tzi ZH, Luo YB (1999). Native orchids of China in colour.

[CR5] Chen X, Gale SW, Cribb PJ, Ormerod P (2009). Anoectochilus Blume. Flora China.

[CR6] Chou LC, Chang DCN (2004). Asymbiotic and symbiotic seed germination of *Anoectochilus formosanus* and *Haemaria discolor* and their F_1_ hybrids. Bot Bull Acad Sin.

[CR7] Govaerts R (2018) World Checklist of *Anoectochilus*. Facilitated by the Royal Botanic Gardens, Kew. http://wcsp.science.kew.org/. Accessed 28 Oct 2018

[CR8] Jiang JH, Lee YI, Cubeta MA, Chen LC (2015). Characterization and colonization of endomycorrhizal Rhizoctonia fungi in the medicinal herb *Anoectochilus formosanus* (Orchidaceae). Mycorrhiza.

[CR9] Kusano S (1915). Experimental studies on the embryonal development in an angiosperm. J Col Agric Tokyo Imp Univ.

[CR10] Lee YI, Lee N, Yeung CE, Chung MC (2005). Embryo development of *Cypripedium formosanum* in relation to seed germination in vitro. J Am Soc Hortic Sci.

[CR11] Lee YI, Yeung EC, Lee N, Chung MC (2006). Embryo development in the lady’s slipper orchid, *Paphiopedilum delenatii*, with emphasis on the ultrastructure of the suspensor. Ann Bot.

[CR12] Lee YI, Yeung EC, Lee N, Lur CF, Chung MC (2007). Changes in endogenous abscisic acid levels and asymbiotic seed germination of a terrestrial orchid, *Calanthe tricarinata* Lindl. J Am Soc Hortic Sci.

[CR13] Lee YI, Yeung EC, Lee N, Chung MC (2008). Embryology of *Phalaenopsis amabilis* var. *formosa*: embryo development. Bot Stud.

[CR14] Lee YI, Hsu ST, Yeung EC (2013). Orchid protocorm-like bodies are somatic embryos. Am J Bot.

[CR15] Miyoshi K, Sato T (1997). Removal of the pericarp and testa of seeds of Japonica and Indica rice (*Oryza sativa*) at various oxygen concentrations has opposite effects on germination. Physiol Plant.

[CR16] Murashige T, Skoog F (1962). A revised medium for rapid growth and bioassays with tabacco tissue culture. Physiol Plant.

[CR17] Nishimura G, Tamura M (1993). Seed coat formation in *Apostasia nipponica*. J Jap Bot.

[CR18] Nishimura G, Yukawa T (2010). Dark material accumulation and sclerotization during seed coat formation in *Vanilla planifolia* Jacks: Ex Andrews (Orchidaceae). Bull Natl Mus Nat Sci Ser B.

[CR19] Pridgeon AM, Cribb PJ, Chase MW, Rasmussen FN (eds.) (2003) Genera Orchidacearum. vol. 3. Orchidoideae (Part 2), Vanilloideae. Oxford University Press, Oxford

[CR20] Rasmussen HN (1995). Terrestrial orchids-from seed to mycotrophic plant.

[CR21] Sato Y (1974). Embryological studies in the Japanese *Epipactis* (Orchidaceae). Sci Rep Tohoku Univ Ser IV (Biol).

[CR22] Yam TW, Yeung EC, Ye XL, Zee SY, Arditti J, Kull T, Arditti J (2002). Orchid embryos. Orchid biology: reviews and persectives VIII.

[CR23] Yamazaki J, Myoshi K (2006). In vitro asymbiotic germination of immature seed and formation of protocorm by *Cephalanthera falcata* (Orchidaceae). Ann Bot.

[CR24] Yang CK, Lee YI (2014). The seed development of a mycoheterotrophic orchid, *Cyrtosia javanica* Blum. Bot Stud.

[CR25] Ye S, Shao Q, Zhang A (2017). *Anoectochilus roxburghii*: a review of its phytochemistry, pharmacology, and clinical applications. J Ethnopharmacol.

[CR26] Yeung EC, Vasil IK (1984). Histological and histochemical staining procedures. Cell culture and somatic cell genetics of plants.

[CR27] Yeung EC (2017). A perspective on orchid seed and protocorm development. Bot Stud.

[CR28] Yeung Edward C., Chan Colin K. W. (2015). The Glycol Methacrylate Embedding Resins—Technovit 7100 and 8100. Plant Microtechniques and Protocols.

[CR29] Yeung EC, Law SK (1992). Embryology of *Calypso bulbosa.* II. Embryo development. Can J Bot.

[CR30] Yeung EC, Law SK, Arditti J, Pridgeon AM (1997). Ovule and megagametophyte development in orchids. Orchid biology: reviews and perspectives VII.

[CR31] Yeung EC, Zee SY, Ye XL (1996). Embryology of *Cymbidium sinense*: Embryo development. Ann Bot.

[CR32] Yeung EC, Li YY, Lee YI, Lee YI, Yeung EC (2018). Understanding seed and protocorm development in orchids. Orchid propagation: from laboratory to greenhouses-methods and protocols.

