# Supplementary figures and images for: Embryology of Anoectochilus roxburghii: seed and embryo development
Source: Bot Stud. 2019 Apr 22;60:6. doi: 10.1186/s40529-019-0254-1 (PMC6476894; doi:10.1186/s40529-019-0254-1)

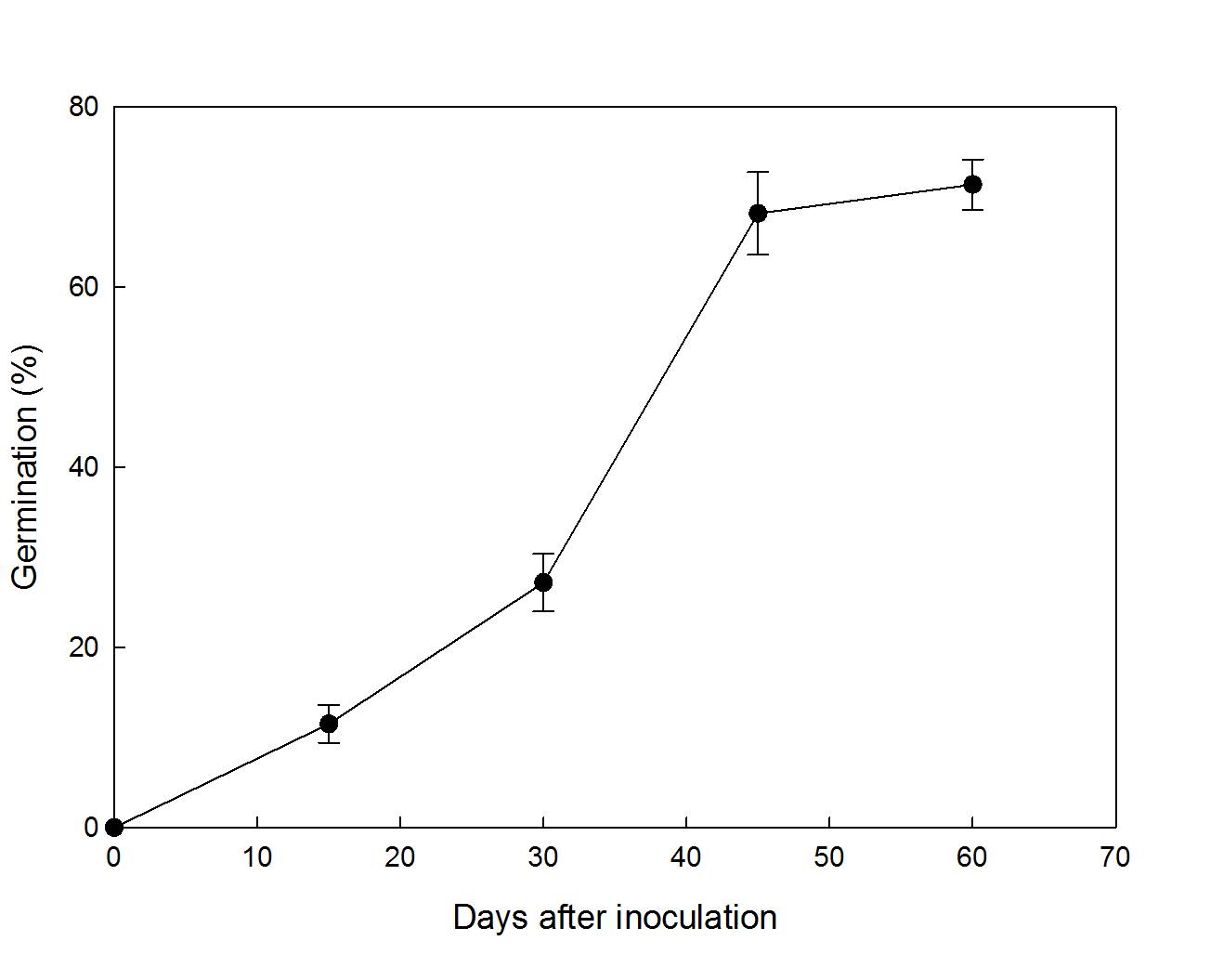

Supplement: Supplementary file 1 — Additional file 1: Figure S1. In vitro seed germination rate of A. roxburghii on 1/4 MS medium. Error bars represent SE (n = 3). [file 40529_2019_254_MOESM1_ESM.jpg]

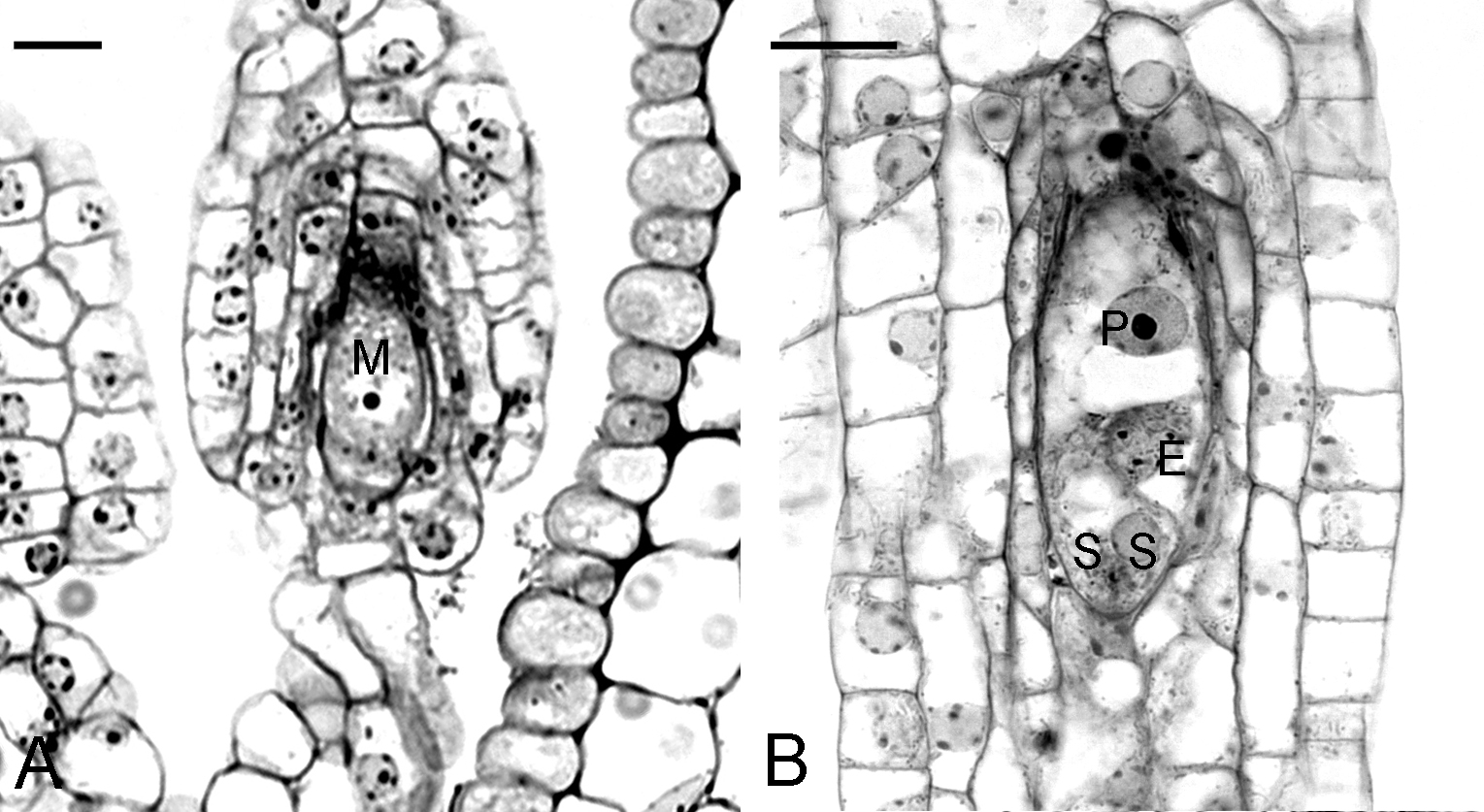

Supplement: Supplementary file 2 — Additional file 2: Figure S2. In A. roxburghii, the megaspore mother cell (M) could be observed within the ovary at the time of anthesis (A), and the mature embryo sac is present at 5 DAP (B). Egg (E), polar nucleus (P), synergids (S). Scale bar = 50 µm. [file 40529_2019_254_MOESM2_ESM.jpg]
